# Periventricular nodular heterotopia in a Chihuahua

**DOI:** 10.1111/jvim.15803

**Published:** 2020-05-23

**Authors:** Leonie F. Herkommer, Manfred Henrich, Christiane Herden, Martin J. Schmidt

**Affiliations:** ^1^ Institute for Veterinary‐Pathology, Justus‐Liebig‐University Giessen Giessen Germany; ^2^ Department of Veterinary Clinical Sciences, Small Animal Clinic, Neurosurgery, Neuroradiology and Clinical Neurology Justus‐Liebig‐University Giessen Giessen Germany

**Keywords:** brain development, dog, malformation, PNH, ventriculomegaly

## Abstract

Periventricular nodular heterotopia is a common neuronal malformation in humans, often leading to epilepsy and other neurologic diseases. A 2‐month‐old female Chihuahua weighing 750 g was examined because of a history of epileptic seizures and abnormalities in gait and behavior. Results of the clinical examination were consistent with a multifocal neurologic disease with localization in the forebrain and spinovestibular system. The magnetic resonance imaging showed multiple bilateral periventricular nodules isointense to gray matter and ventriculomegaly. Histopathological and immunohistological examination of the brain revealed that periventricular nodules consisted of neurons, fewer astrocytes, and some oligodendroglia consistent with periventricular nodular heterotopias.

**Abbreviations**
FLN1: filamin 1
MRI: magnetic resonance imaging
PNH: periventricular nodular heterotopia

## 1 INTRODUCTION

Gray matter heterotopias describe a group of migration disorders in which neuronal cells fail to migrate normally during development of the cerebral cortex.^1^ Early in the formation of the cortical laminae, neuronal precursors align at the border of the lateral ventricles. In this ventricular zone, the cells multiply, start to migrate radially with the help of radial glia toward the pial surface and settle in a thick primordial cortex layer, the so‐called cortical plate. Each new cohort of neurons migrates past the settled cortical plate neurons until ultimately a six‐layered cortex is built. Errors in neuronal migration can lead to different forms of ectopic clusters of neurons, which are in sum referred to as heterotopias.^1^, ^2^ Gray matter heterotopias can be subdivided into 3 groups. Subcortical heterotopias are nodular or curvilinear masses of gray matter, which protrude into the white matter while being connected to the overlying cerebral cortex.^3^ Band heterotopias, also called double cortex, on the other hand are layers of gray matter that lack any connection to the cortex.^3^ In periventricular nodular heterotopias, nodules of gray matter are found unilaterally or bilaterally in close proximity to the lateral ventricles, protruding into the lumen or lining the ventricular walls.^4^ This case report describes the clinical signs and magnetic resonance imaging (MRI), histological, and immunohistological findings of periventricular nodular heterotopia (PNH) in a dog.

## 2 RESULTS

### 2.1 Clinical findings

A 2‐month‐old female Chihuahua weighing 750 g was examined because of a 4‐week history of abnormal behavior, gait abnormalities, and generalized tonic‐clonic seizures, which occurred every 24 to 48 hours with a duration of 2 to 5 minutes. Neurologic examination confirmed the complaints and revealed circling to the right as well as ataxia on all 4 limbs. Postural reaction deficits were identified in all 4 limbs. The menace response was absent from both eyes with normal pupillary light reflexes. A mild ventrolateral strabismus was present in both eyes. There was a positional horizontal nystagmus in both eyes in dorsal recumbency and no reaction to a falling cotton ball. Segmental spinal reflexes were normal. Clinical findings were compatible with a multifocal localization including the forebrain and the spinovestibular system.

General physical examination, complete blood count, biochemistry panel, and electrolyte examination were within the normal limits for a dog that age with normal serum bile acids and blood ammonia concentrations.

### 2.2 MRI findings

An MRI of the brain was performed with a 3.0 Tesla superconductive system (Siemens Verio) and sensitivity‐encoding coil. Sagittal, dorsal, and transverse T2‐weighted(TR/TE = 2900/120 [ms]), transverse FLAIR sequences (TR/TE = 7000/120 [ms], TI = 2400 ms,), and transverse T1‐weighted sequences (TR/TE = 491/8 [ms]) before and after administration of a gadolinium‐based contrast agent were acquired. Slice thickness was 2 mm, FOV 180 × 180 mm, with a matrix of 288 × 288.

**FIGURE 1:**
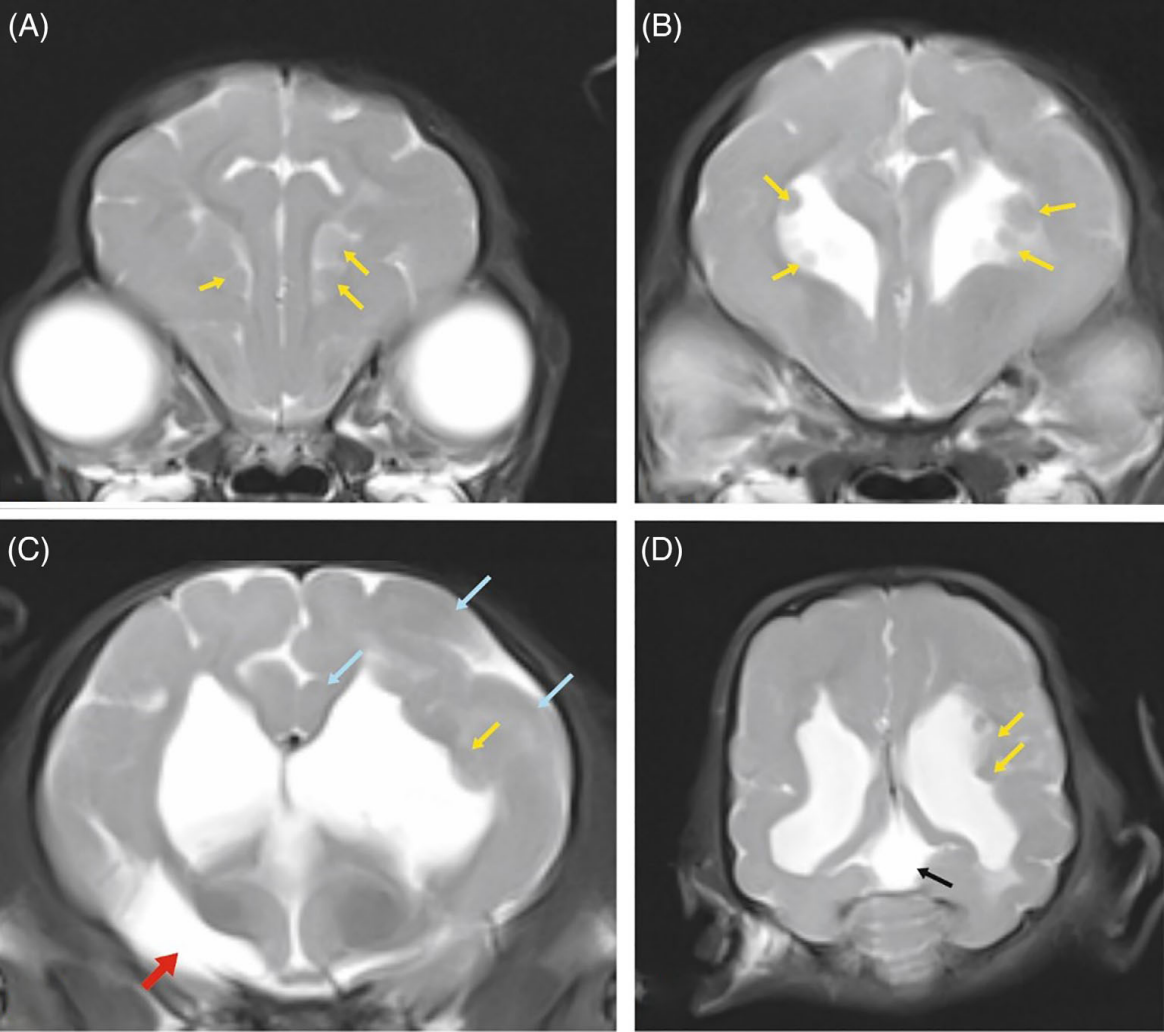
A, B, C, Transversal and D, dorsal T2‐weighted magnetic resonance images of a Chihuahua. Single round to ovoid nodules (yellow arrows) line the inner surface of the lateral cerebral ventricles on both sides. The nodules have the same signal intensity as the overlying gray matter. The gyrification pattern is irregular with smaller and larger gyri compared with the other hemisphere (blue arrow). There is bilateral symmetrical ventriculomegaly of the lateral cerebral ventricles and a supracollicular fluid accumulation (black arrow). The corpus callosum is thin, C. There is a large irregular parenchymal defect in the right ventral thalamus filled with cerebrospinal fluid (red arrow)

MRI revealed aberrations from normal brain anatomy (Figure 1). The lateral cerebral ventricles were subjectively enlarged. In the rostral horn and body of both lateral cerebral ventricles, there were multiple small round to ovoid lesions adjacent to the periventricular white matter elevating and distorting the ventricular outlines. The lesions were isointense to gray matter in all sequences. In the ventral parenchyma of the right hemisphere, there was an irregular hyperintense lesion in T2‐weighted and FLAIR images extending from the caudate nucleus and putamen caudally toward the thalamus and amygdala. The same lesion was hypointense in T1‐weighted images without contrast enhancement and was consistent with an encephaloclastic defect. The gyrification pattern was abnormal with multiple irregular larger and smaller gyri with asymmetries between the left and right hemisphere. Gray‐white matter contrast was low as expected for a young dog. The midbrain, cerebellum, and medulla had a normal morphology and myelination. Based on these findings, extensive malformation of the cerebral hemispheres was diagnosed, including bilateral periventricular nodular heterotopias, ventriculomegaly, pachygyria, encephaloclastic defects in basal nuclei, and a supracollicular fluid accumulation (Figure 1).

### 2.3 Histopathologic findings

The dog was euthanized and the brain was cosmetically exenterated and brain samples were submitted for a postmortem examination. Three‐micrometer‐thick slices of formalin‐fixed and paraffin‐embedded brain tissue were stained with hematoxylin and eosin, cresyl violet, and Luxol Fast Blue stain. Immunohistochemical staining was performed with antibodies against neuronal nuclei (NeuN, Chemicon, monoclonal mouse antibody, diluted 1:100), neurofilament (Dako, monoclonal mouse antibody, diluted 1:400), synaptophysin (Dako, monoclonal mouse antibody, diluted 1:100), and glial fibrillary acidic protein (GFAP, Dako, polyclonal rabbit antibody, diluted 1:500).

**FIGURE 2:**
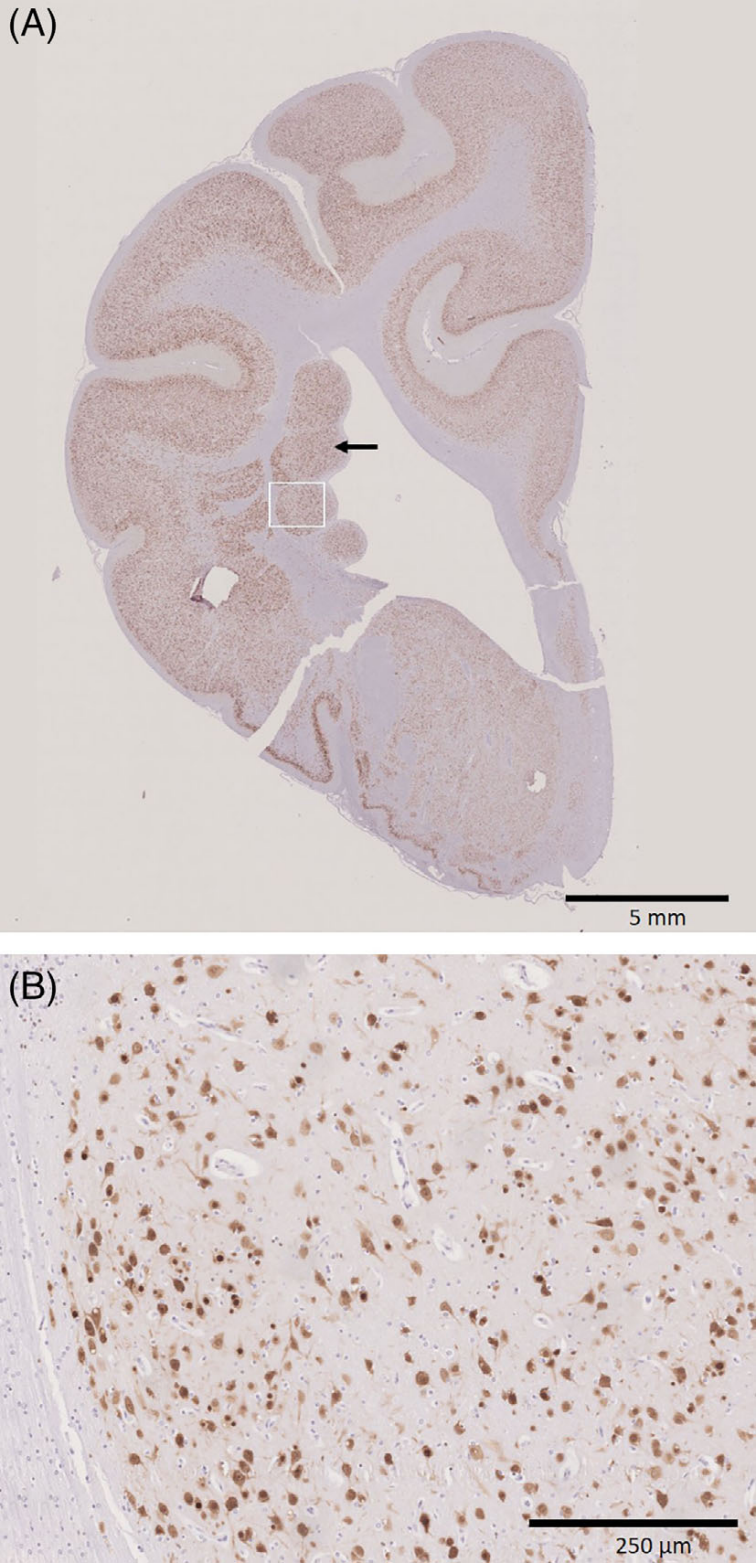
Immunohistochemistry with anti‐NeuN antibody. A, Coronal section of the right hemisphere showing periventricular nodules (arrow) with NeuN positive neurons (×0.5). B, Insert, Higher magnification view of the periventricular nodules with haphazardly distributed NeuN positive neurons (×10). NeuN, neuronal nuclei

**FIGURE 3:**
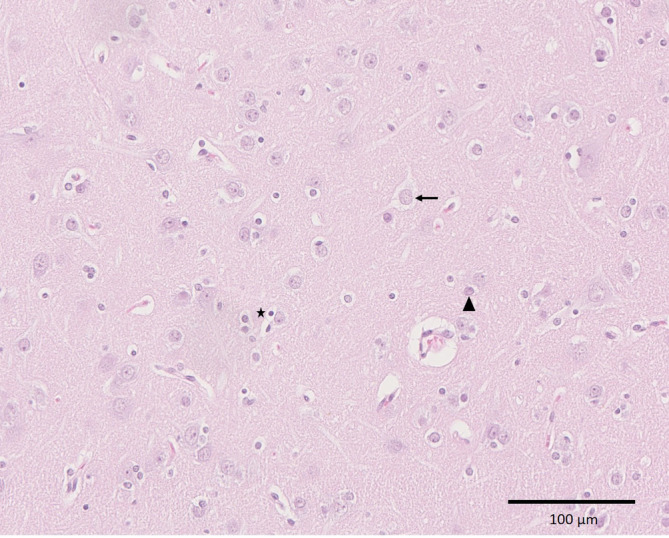
Hematoxylin and eosin stained section of the heterotopic nodules resembling normal gray matter with relatively large pyramidal‐shaped neurons (arrow), astrocytes (arrowhead), and oligodendroglia (asterisk) (×20)

**FIGURE 4:**
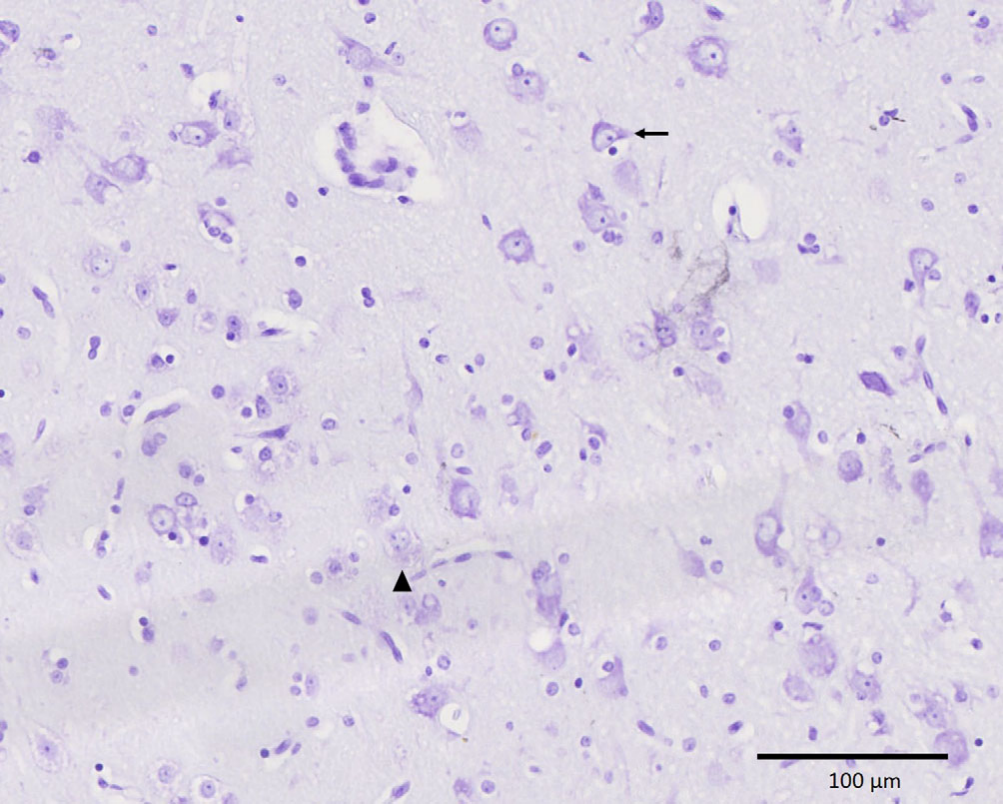
Cresyl staining of the heterotopic nodules showing an irregular distribution of Nissl substance with some intensely (arrow) and some palely stained neurons (asterisk) (×20)

**FIGURE 5:**
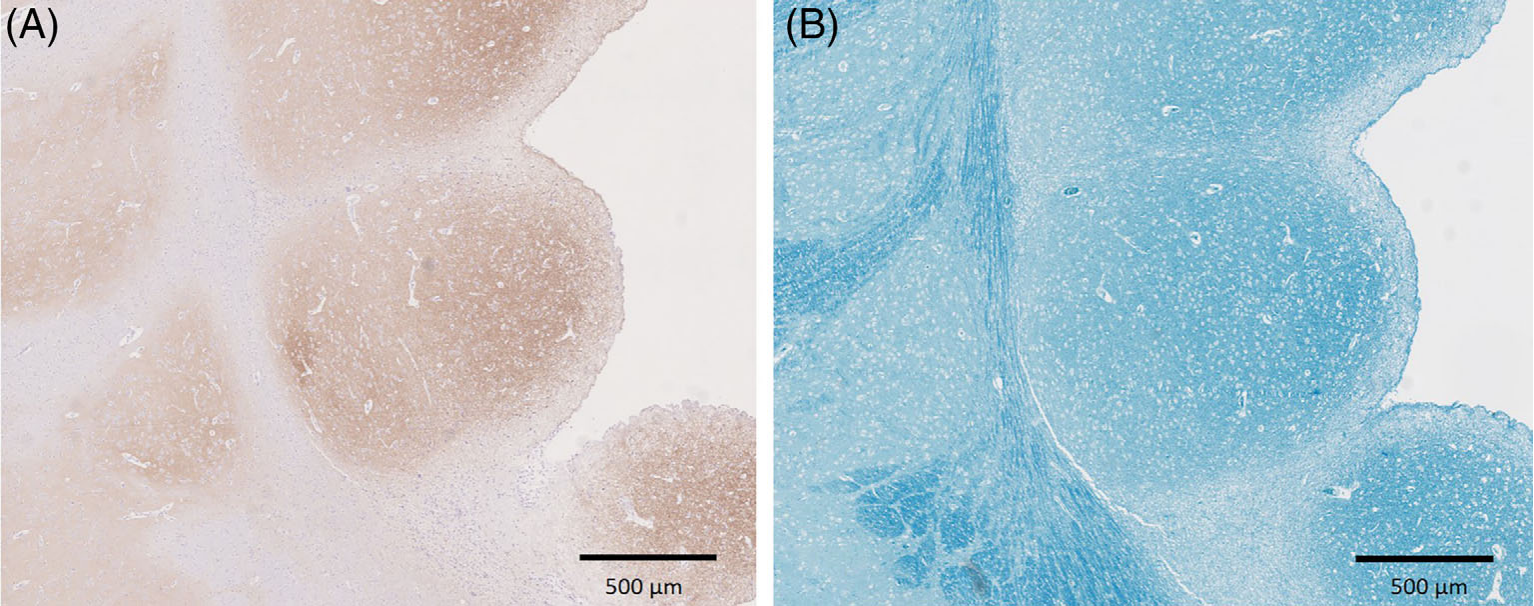
Nodules are separated from each other, from the ventricular ependyma and from the adjacent white matter and striatum by a poorly cellular matrix neither consistent with gray matter, A, Synaptophysin negative, nor white matter, B, Luxol negative (×3)

The histological features of the nodules, which were isointense to gray matter in the MRI, were consistent with cases of PNH as described in humans.^5^, ^6^, ^7^ On histological examination the periventricular nodules consisted of well‐differentiated, haphazardly distributed neurons (NeuN positive), astrocytes (GFAP positive), and oligodendroglia (Figures 2 and 3). Their neuronal nuclei were mostly about 15 μm in diameter with a finely stippled chromatin and 1 or 2 inconspicuous nucleoli intermingled with a few small neurons containing smaller nuclei measuring about 7 to 10 μm. The neurons had a moderate amount of pale basophilic cytoplasm and large basal and apical projections with variable orientations. Cresyl violet stain revealed irregular distribution of Nissl substance with a few intensely and a few pale stained neurons (Figure 4). Unlike the description of human cases, there were only a few neurofilament‐positive fibers within the nodules^7^ and no glomerular‐like changes of the blood vessels.^5^ Blood vessels within the nodules showed no histological alterations. Nodules were separated from each other, from the ventricular ependyma and from the adjacent white matter and striatum by a poorly cellular matrix neither consistent with gray matter (synaptophysin negative) nor white matter (Luxol fast blue negative) (Figure 5).

**FIGURE 6:**
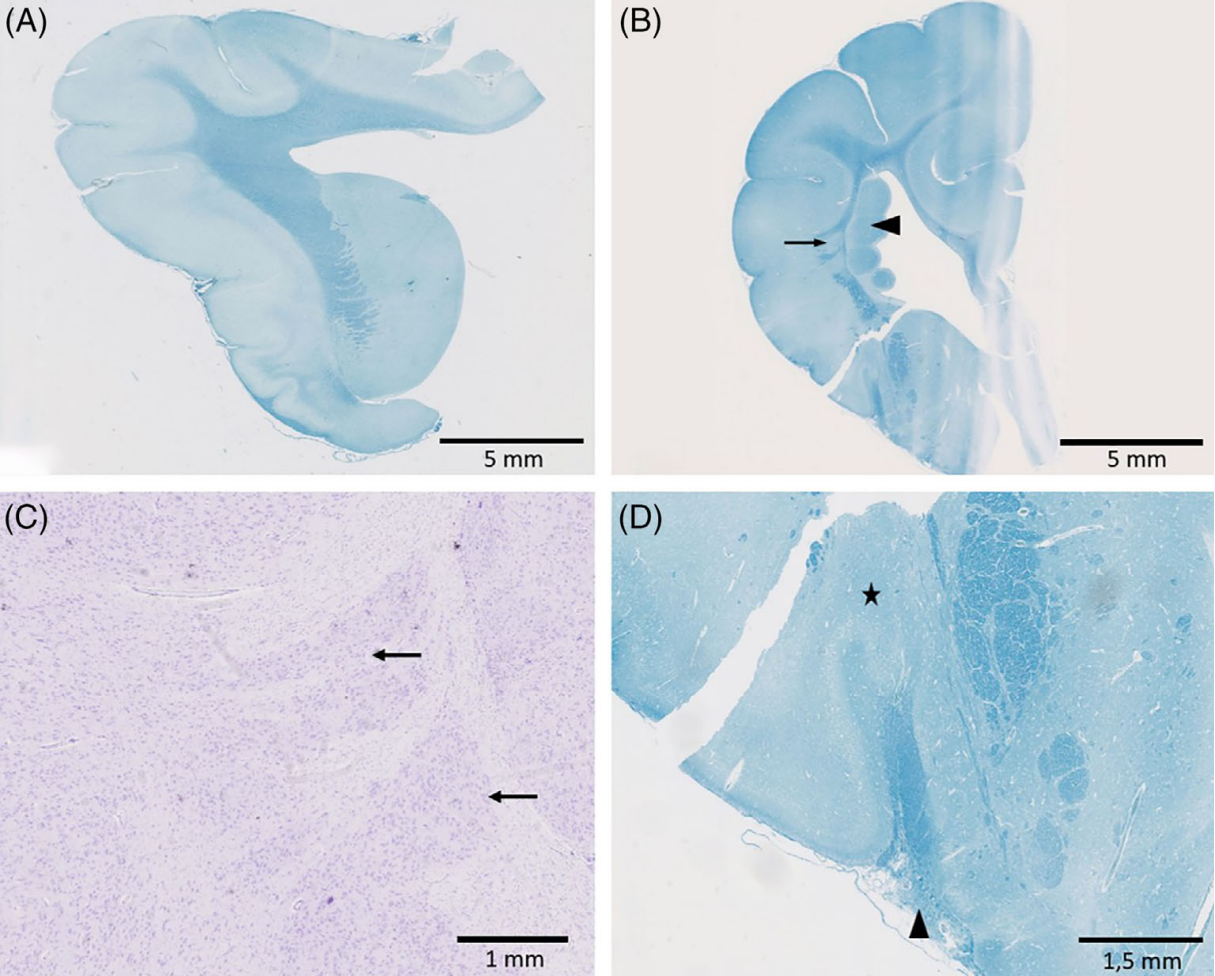
A, Section of an age matched Chihuahua with ventriculomegaly (Luxol, ×0.5). B, Present case, 2 groups of neurons (arrow) without connection to the heterotopic nodules (arrowhead) (Luxol, ×0.5). C, Higher power view of the groups of neurons with a cortex‐like arrangement (arrows) (cresyl, ×1.5). D, Focal meningothelial proliferation in close proximity to the rhinal fissure (arrowhead) and a nearly indistinguishable external capsule (asterisk) (Luxol, ×1)

Lateral to the nodular heterotopias there were 2 groups of neurons with connection to the overlying cortex (Figure 6B). Their arrangement was comparable to cortical architecture (Figure 6C). The nucleus caudatus was small and displaced ventrally with an almost indistinguishable external capsule (Figure 6D). The meninx overlying the rhinal fissure showed mild focal meningothelial proliferation (Figure 6D). A moderate number of periventricular glial progenitor cells was found in close proximity to the nodules, some of them inside the striatum. The pachygyria and ventriculomegaly detected on the MRI were also visible in the histological slices. In the medulla oblongata and in the ventral portion of both hippocampi there were a few hypereosinophilic neurons indicating neuronal degeneration. There was mild hypoplasia of the corpus callosum. Hippocampal sclerosis, as described in human cases of PNH,^8^, ^9^ was not evident. The lamination of the cerebral cortex was normal.

## 3 DISCUSSION

Focal cortical dysplasias describe a group of heterogeneous malformations that have already been reported in animals, albeit rarely.^10^, ^11^, ^12^ Studies on heterotopias are limited to a cat,^13^ a Lagotto Romagnolo dog (Rusbridge C, Wilkins P. Neuronal heterotopia in a Lagotto Romagnolo dog [Abstract]. *Neuropath Appl Neuro*. 2002;28:162‐162), an unpublished and not further described case of a suspected subependymal nodular heterotopia in an adult miniature dachshund as part of an epilepsy study,^14^ a case of a miniature schnauzer where the authors noted mild heterotopia of neurons^15^ and a sealion.^16^ Here we describe clinical signs, MRI, and histopathological findings in a Chihuahua with PNH. Neuronal heterotopias are a manifestation of disruption of normal neuronal migration. In humans, the conglomeration of ectopic cells range in size from small, discrete neuronal clusters, which seems to be the most common form,^2^ to large multinodular conglomerates, occurring as focal or multifocal masses in the hemispheres.^1^, ^2^, ^3^ In a more generalized form of PNH, bilateral contiguous nodules create an irregular bumpy surface of the whole ventricular wall.^1^, ^2^, ^3^ Based on the location of the ectopic cells, heterotopias can be characterized as subependymal, subcortical, and band heterotopias.^1^, ^2^, ^3^ In a cat^13^ and a sea lion^16^ that were diagnosed with heterotopia, ectopic neurons presented as large irregular confluent nodules of gray matter that replaced physiological architecture of the thalamus and cerebral hemispheres. Both were classified as subcortical heterotopias.^13^, ^16^ The morphological form in the Chihuahua in this report rather resembles typical PNH consisting of small noncontiguous bilateral nodules underneath the ependyma of the lateral ventricles.^3^

Not least because of the rarity of heterotopias in animals, the pathogenesis of the disorder is largely unknown. In pregnant rats, gray matter heterotopias were experimentally induced in the offspring by treating the dam with radiation^17^ or by injection of the neurotoxin methylazoxymethanol.^18^ Cocaine exposure to pregnant mice and monkeys has also been shown to disturb neuronal migration.^19^ In humans, PNH has already been associated with a variety of chromosomal copy number variations.^20^, ^21^ Furthermore, an X‐linked genetic defect in the filamin 1 (FLN1) gene was found.^22^ FLN1 helps to build up and maintain the intracellular actin cytoskeleton as well as the extracellular network of actin filaments.^23^ Defects in FLN1 proteins do not only cause abnormal neuronal motility, but also a loss of structural integrity of the neuroepithelial cells in the ventricular zone, causing disengagement of the neurons and the development of ectopic cells.

Interestingly, lamination of most of the remaining cerebral cortex is largely preserved in humans with PNH.^24^ However, heterotopias can be associated with other congenital brain abnormalities including, internal hydrocephalus, cerebellar hypoplasia, corpus callosum anomalies, arachnoid cysts, polymicrogyria, and a spectrum of caudal fossa malformations.^2^, ^3^ A teratological connection between the PNH and the other malformations in this case could be possible, however, was not proven. Moreover, in none of the human cases is there an association between heterotopias and dysplasia of the caudate nucleus. Because the nucleus caudatus was hypoplastic in the present case, it could be hypothesized that the heterotopic neurons might have been arisen from the caudate nucleus. The meningothelial proliferation in the area of the rhinal fissure could indicate a reactive process and might therefore underline the theory of an encephaloclastic defect. Detailed neuropathological investigation revealed shrunken neurons in the ventral part of both hippocampi possibly as a result of acute seizures.

In human PNH cases with genetic FLN defects, extraneural malformations are also described. FLN1 is required for cell adhesion and fusion, for example, in the development of the heart and other midline structures. Therefore, patients with PNH can also show cardiac abnormalities including atrial septal defects, bicuspid aortic valve, and patent ductus arteriosus.^25^ FLN1 function also induces platelet aggregation by coupling thrombin and von Willebrand's factor receptor.^26^ Abnormalities of this critical function can contribute to both, hypercoagulability and hemorrhagic disorders in humans. Furthermore, FLN1 gene defects induce contraction of vascular smooth muscle cells.^27^ Both of these malfunctions are implicated to predispose for strokes at an extremely young age, which can also be a comorbidity in humans with PNH but has not been described for animals so far. Affected patients show heterogeneous clinical signs depending on the spectrum of associated malformations and comorbidities.^28^ They range from mild drug responsive epilepsy and normal intelligence to refractory epilepsy, severe mental retardation, and early postnatal mortality.^29^ In general, the exact mechanism of PNH for the generation and propagation of epileptic discharges is not fully elucidated.^30^ Seizures are present in the majority of patients with PNH, but not all of them, and the occurrence of seizures is neither related to the size nor the localization of heterotopias.^28^, ^30^ Electroencephalographic recordings show the involvement of the heterotopic nodules in epileptic discharges^31^ and it is likely that seizures are generated by abnormal anatomic circuitries including the heterotopic nodules, the hippocampus, and neocortical areas.^30^ This hypothesis is further supported by the positive results of surgical resection of the PNH on seizure activity.^6^, ^32^ Despite widespread alteration of normal brain morphology, the only clinical signs in animals with PNH reported so far were seizures. However, subtle neurological deficits and especially cognitive impairment cannot be properly assessed in animals and the real status of physiological brain functions cannot be determined with absolute certainty. The Lagotto Romagnolo dog was also presented with gait abnormalities, which were, unfortunately, not further characterized (Rusbridge C, Wilkins P. Neuronal heterotopia in a Lagotto Romagnolo dog [Abstract]). Circling behavior, ataxia, and disturbances of physiological eye movement in our case were most likely because of the encephaloclastic defect in the striatum and medioventral thalamus in the Chihuahua possibly as a residual lesion because of an intrauterine infarction.

In summary, gray matter heterotopia should be included as a rare differential diagnosis in young dogs suffering from seizures that can be clinically diagnosed by MRI. Genetic investigation for FLN1 gene defects in animals with PNH might be of interest.

## CONFLICT OF INTEREST DECLARATION

Authors declare no conflict of interest.

## OFF‐LABEL ANTIMICROBIAL DECLARATION

Authors declare no off‐label use of antimicrobials.

## INSTITUTIONAL ANIMAL CARE AND USE COMMITTEE (IACUC) OR OTHER APPROVAL DECLARATION

Authors declare no IACUC or other approval was needed.

## HUMAN ETHICS APPROVAL DECLARATION

Authors declare human ethics approval was not needed for this study.
